# An overview of the health care system in Georgia: expert recommendations in the context of predictive, preventive and personalised medicine

**DOI:** 10.1186/1878-5085-4-8

**Published:** 2013-02-26

**Authors:** Tamari Rukhadze

**Affiliations:** 1 Faculty of Medicine, Iv. Javakhishvili Tbilisi State University, 2 Chiaureli st, Tbilisi 0179, Georgia; 2 National Cancer Centre of Georgia, Lisi Lake, Tbilisi 0177, Georgia

**Keywords:** Georgia, Health care system, Primary health care, Hospital sector, Strategic plan, Predictive, Preventive and personalised medicine

## Abstract

The main aim of this paper is to present the current statistics and situation of health care system in Georgia; the changes in the transition period within the society and the health care system. Also presented are the efforts from the Government and the Ministry of Labour, Health and Social Affairs of Georgia in the way of numerous initiatives and action in order to improve quality care of patients and sustain the health care system. This paper described the institutional framework, process, content and implementation of health and health care policies in Georgia in the context of predictive, preventive and personalised medicine.

## Review

### Introduction

Georgia is a sovereign state in the Caucasus region of Eurasia, situated in the South Caucasus and is bounded to the west by the Black Sea, to the north by Russia, to the south by Turkey and Armenia, and to the southeast by Azerbaijan.

### Historical introduction

According to the literature sources, the Semashko model of health care was adopted during the Soviet period (1921–1991) in Georgia as other Soviet countries. After the collapse of the Soviet Union, Georgia’s population decreased by nearly a fifth and the economy rapidly moved from a communist regime to a market system [1-3]. Real per capita public expenditures on health care rapidly declined from around US $13.00 in 1990 to less than US $1.00 in 1994 [1,4,5]. Data shows that in the 1990s, social health insurance as a mandatory was introduced. It has been abandoned, and private health insurance is being promoted as the main mechanism for the pre-payment of health services in Georgia.

Planning for health care reform was reported from 1993, led by the Ministry of Labour, Health and Social Affairs of Georgia (MoLHSA) and undertaken during the post-independence shift towards a market economy. The first major changes took place as a result of the 1995 Georgian Health Care Reform Package that introduced new concepts, including social insurance, official user fees and new provider payment mechanisms like co-payments [1,2,4]. Data shows that in 1999, the Georgian National Health Policy, which outlined objectives to improve the equity, accessibility and affordability of health services, was developed [1,2].

Georgia has made a significant effort to adapt health policy and the health system to the new environment. Private insurance coverage for households living below the poverty line is paid from public funds, but all other individuals are expected to purchase cover on their own initiative. Out-of-pocket payments remain the main source of funding for the health system in Georgia, which reduce access to services for much of the population, particularly in access to pharmaceuticals. Overall, health system regulation is rather weak, particularly when compared with the challenges it faces [2,4-6].

### Demographic situation

#### Life expectancy

**Figure 1:**
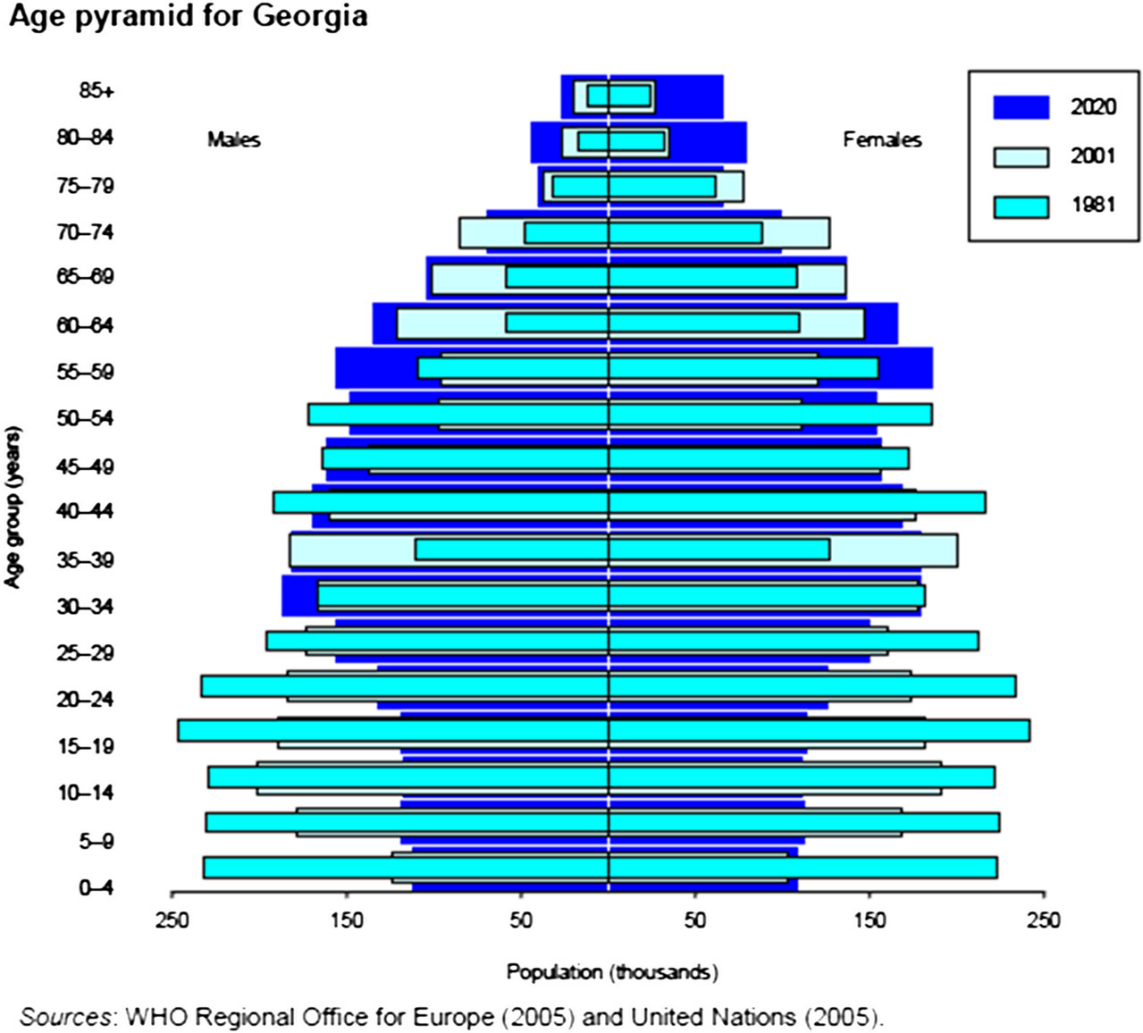
Age pyramid of Georgia.

According to the data of health report of MoLHSA and National Centre for Disease Control and Public Health (NCDCPH), the life expectancy in Georgia is 69.2 for males and 77.7 for females. The age pyramid for the population of Georgia presented by World Health Organisation (WHO) regional office for Europe and United Nations is presented in Figure 1. For the past several years, life expectancy has been slowly growing in Georgia and reached 73.6 years in 2010 for males and females, which indicates an improvement in the health of the population [4,7-11].

#### Fertility and mortality rates

The MoLHSA and NCDCPH estimate that in 2009, the average annual number of population was approximately 4,410,900, and by January 1, 2010, this number has been increased by 4,436,400. Of the population, 52.5% were women; 47.5%, men. By the end of 2009, health report published 63,377 live births, where 32,385 (51.1%) were males and 30,992 (48.9%) were females. It is reported that in 2009, the number of female live birth increased within 5%. The demographic development at all stages of the rural population was characterised by a high birth rate, and according to the same report in the last decade, the rate reduced. In 2009, the only 31.1% from all live births were rural inhabitance. The official report of MoLHSA and NCDCPH represented that the infant mortality rate in 2005–2007 was reduced per 1,000 live births, but in 2008, the rate was increased and reached to 17.0. The large discrepancy exists between rural and urban mortality rates among children under 5 years and almost twice many deaths have been registered per 1,000 live births in rural areas than in urban areas [4,7,12-16].

In 2010, infant mortality rate was15.7 per 1,000 live births, and the children mortality rate under 5 years was 17.1. According to the statistics, by the 2050, infant mortality rate is expected to drop to 5.6, and the children mortality rate under 5 years to drop to 6.2 [5,8,10]. Since 1989, significant decrease in birth and natural growth rates was noted in Georgia. From 2006 to 2008, the tendency of increasing both indicators was noted.

#### Disease and leading causes of death

According to the evaluation of WHO experts in 2002, the disease burden of non-contagious disease was 89%, contagious disease was 5% and disease burden of trauma was 6%. The third place from the top ten reasons of disease burden is represented by conditions caused in the prenatal period. In 2002, the top seven causes of disease burden in children aged 0–14 (disability-adjusted life year per 1,000 child) was evaluated by the WHO experts [5,7,8,10]. The first place presented the following pathologies: low body weight at birth, birth asphyxia and maternity traumas—total of 32.4%; the second place, the upper and low respiratory tract infections (7.1%); and the third place, other congenital abnormalities (1.7%) (cardiac abnormalities, Down’s syndrome and spina bifida). Maternal mortality was reduced from 70 and was 14.3 per 100,000 live births. According to the sudden death syndrome, infant mortality rates have been decreased from 23.7 to 14.3.

### Health care system in Georgia

The State Commission for Regulating Social Policy was set up as part of the 2000–2009 strategic health plan of Georgia. It reported directly to the president and provided guidance to the MoLHSA, the National Health Management Center, Regional Health Departments and other health-related sectors [1,2]. The role of the National Health Management Center was determined to provide scientific and technical input into the process of health sector reform, and it worked directly with both international and local non-governmental organisations [1,2,4,17]. The MoLHSA manages the public health services, and the role of government in regulating health care and financial transactions has been greatly reduced since 2003 [2,5].

According to the financial records, Georgia also receives substantial external health financing from sources such as the United Nations, the World Bank, non-governmental organisations and other countries, including Germany, Japan, the United Kingdom, and the United States [1,17-19].

The Governmental Commission for Health and Social Reforms, the State Minister of Public Reforms, and MoLHSA developed ‘Mein Directions in Health 2007–2009’, which outlined the 3-year health sector transformation. It focused on ensuring affordability, quality, accessibility and efficiency on health services. It also introduced market-based principles to health care management [3,12,13]; about 80% of the hospitals were sold to the private sector for redevelopment as modern and most of them as multi-profile hospitals. Nearly all health care providers are private actors, independent of the state.

**Figure 2:**
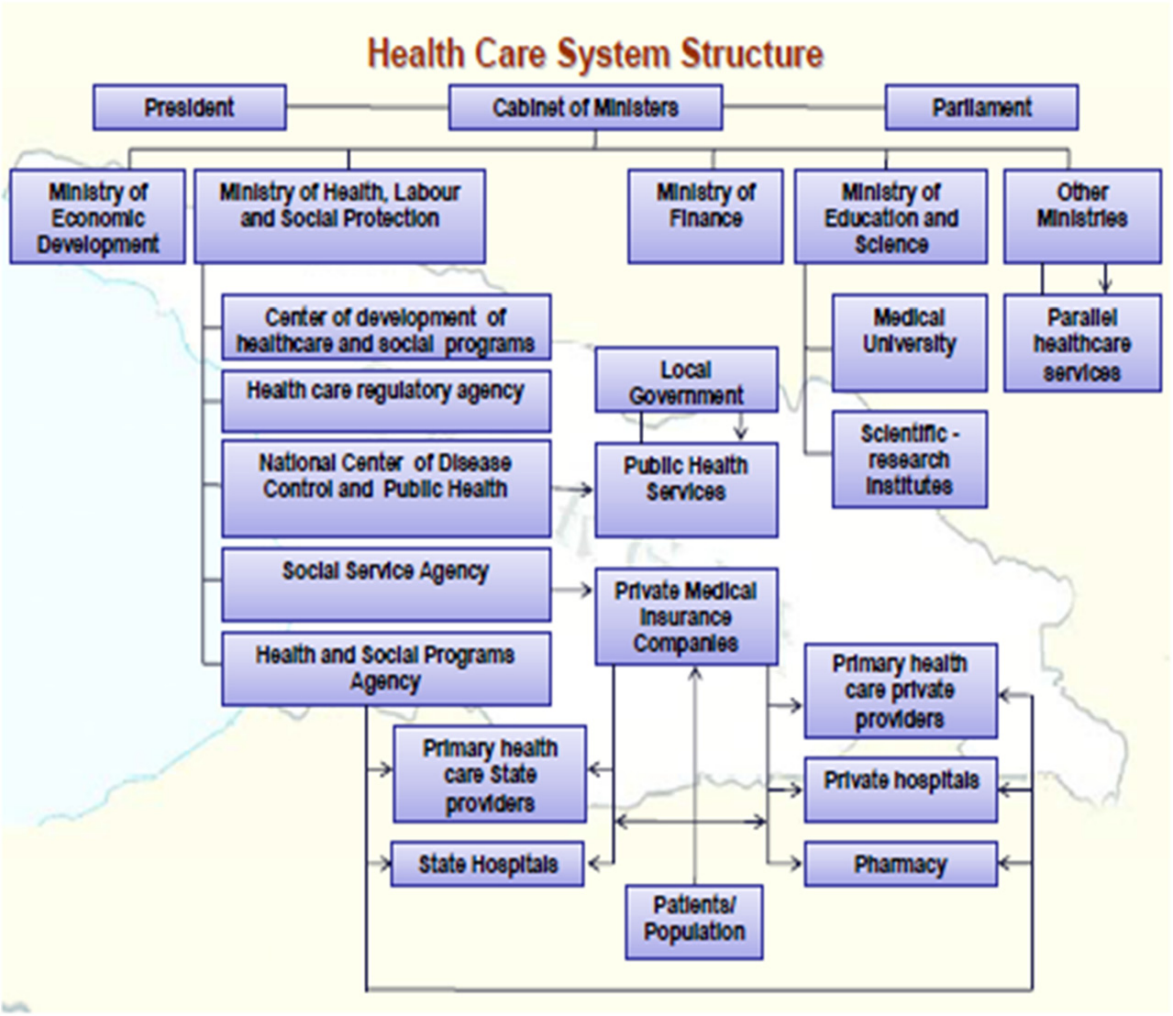
**Health care system structure in Georgia by 2009.** Adapted from Chanturidze et al. [2] and Kordzaia [21].

Currently, the National Healthcare Policy is still under development in Georgia [7,20,21]. The official data of 2009 shows that health care and health insurance are privatised, and organisational structure of health care system can be depicted in Figure 2.

Regardless of the complexity of Figure 2, three principal fields closely linked to each other can be classified within the Georgian health care system:

1. Public health

2. Primary care setting

3. Hospital sector [21]

### Public health

Transformation of soviet sanitary—epidemiologic surveillance into public health system, which started in 1996—is still lingering on the level of undefined logical framework, unable to make development perspectives for the field more or less predictive. Conceptual scheme for structuring Georgian public health field, bordering the competences, objectives, legal rights and obligations are defined in the Georgian law ‘on Public Health’ adopted in 2007 [2,7,21].

According to the law, public health is defined as the complex of measures directed towards health improvement, disease prevention and control in the population, which aims at supporting the general welfare and healthy lifestyle of the population, providing healthy and safe environment for living, bolster the reproductive health and preventing from both contagious and non-contagious disease outspread. The above mentioned shows that predictive, preventive and personalised medicine (PPPM) elements are increasingly becoming a recognised concept and should be gradually integrated into health care systems [11,16].

The literature described that according to the law, the subjects pertaining to the public health field competence involve the following: prevention of transmittable diseases, identification of transmittable diseases, isolation and putting a person to quarantine, provision of biological safety, setting norms of air, water and earth composition, sound, vibration and EM radiation limits and surveillance, chemical, radiation, technology and production safety, defining healthy lifestyle, maternal, child and adult health policy, control on tobacco consumption, drug addiction, toxicomania and alcoholism and taking respective measures against the above listed [12,13,21].

Launching the law on Public Health implicates the delegation of competences on different ministries and local governances within the public health field (including Ministry of Agriculture, Fisheries and Food, Ministry of Environmental Safety and Natural Resources, Ministry of Education and Science, Ministry of Internal Affairs, Ministry of Defense, and Ministry of Justice) and coordination of public health policy making and implementation assigned to the MoLHSA.

The deemed structure of public health can be formulated like the following:

1. Policy and strategy in defining the structure in the central apparatus of the Ministry

2. Strategy in implementing, coordinating, monitoring and analysing the structure in the capital and local regions (National Center of Disease Control and its office)

3. Financial means needed for the implementation of educational, expert and research activities, implying state programmes [10,13,21]

### Primary care setting and hospital sector

Data shows that the course of training doctors (especially therapeutic field physicians) into general practitioners and remaking polyclinic system into family medicine centres was initiated by the end of the twentieth century in Georgia. This process has been progressing slowly with some hindrances due to different reasons, resulting in simultaneous activity of both systems in today’s health care system, with more or less similar competences. Polyclinics and family medicine centres coexist both in the cities (including the capital) and regional centres as well. The system of rural doctor and nurse also remains the main health care provision in the villages, composing the primary care setting for the village-dwelling population. The rural medical personnel refers to the village outpatient setting and is related to regional family medicine centre or polyclinic under agreement, which provides the specific monitoring for their professional activity and assists in the management of complex cases. For the past several years, up to 50 modernly equipped hospitals have started functioning [12-14,16,21].

#### Health service capacity and health care financing

Since 1990s, different reforms have been done to manage the system at the central, regional and municipal levels. In the scale of changes during the last years, the government of Georgia with different international organisations focused on the development of primary health care and reduced the dominancy of hospital projects.

Enrolled as a wholly tax-funded health care system in 1990s, reforms in 1995 replaced this system with a social insurance model run through the State Medical Insurance Company. Under the social insurance model, basic health care was paid for by the state insurance company, with additional funds coming from municipal health funds and preventive activities provided by the MoLHSA [1,19,22].

**Figure 3:**
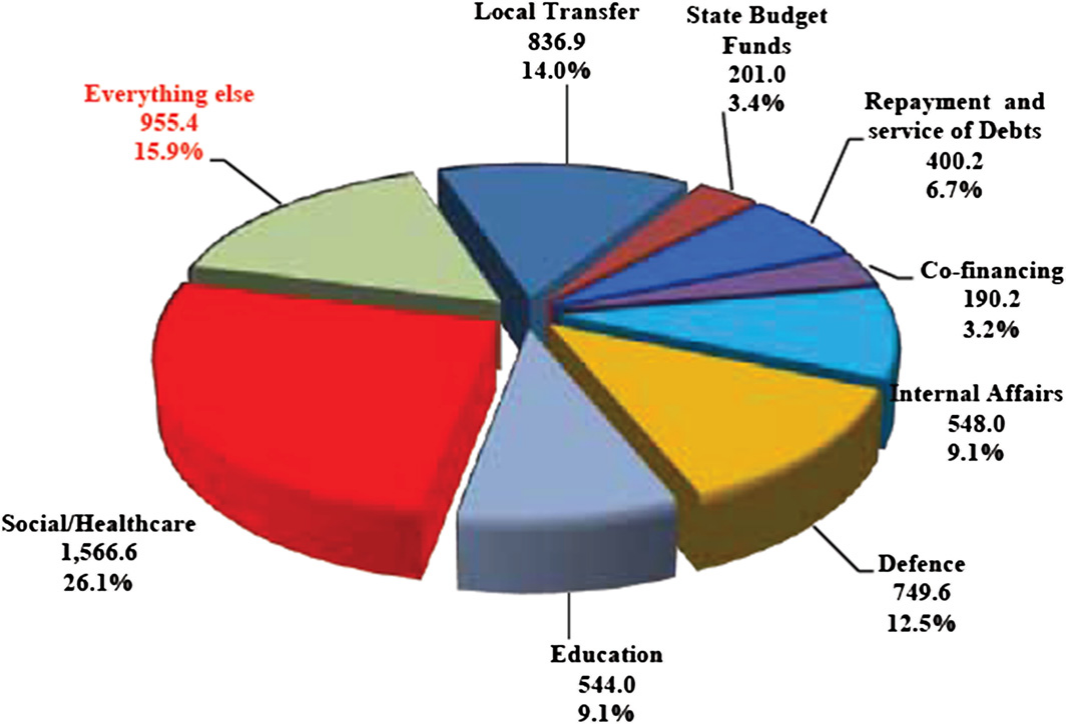
The total budget breakdown (Source - A Citizen’s Guide to the 2010 State Budget of Georgia. Report of Ministry of Economics. 2011).

The major breakdown of the total budget is presented for the year 2010 and represented by the Ministry of Economics in the Citizen’s Guide to the 2010 State Budget of Georgia (see Figure 3).

Total health expenditure as a percentage of gross domestic product (GDP) in 2007 fell back to 8.2%, but public health expenditure on health was only 1.5% of GDP and 4.7% of general government expenditures in 2007. According to the publication presented by WHO, it is estimated that total health expenditure as a percentage of GDP in Georgia reached 8.6% in 2005, which is relatively high in international comparisons, the EU average being 8.9% while the Commonwealth of Independent States (CIS) average was 5.5% for the same year [2,18,19,22].

**Figure 4:**
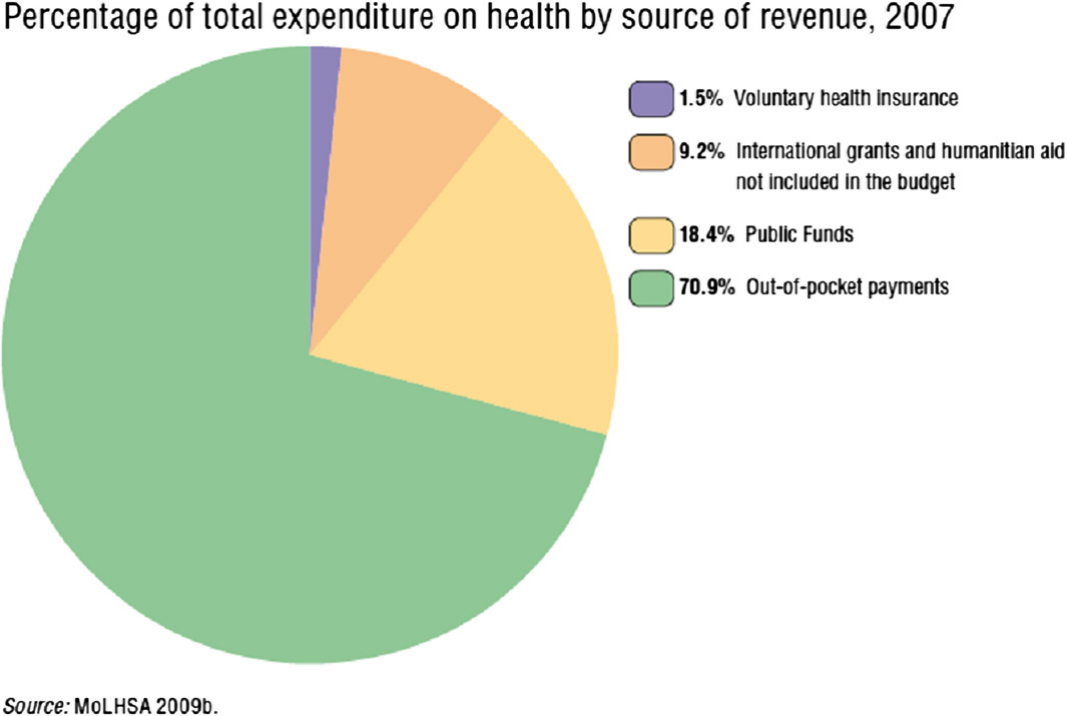
Percentage of total expenditure on health by source of revenue, 2007 presented by T. Chanturidze and all., 2009.

Total health expenditure has been rising in Georgia since the late 1990s, which is in marked contrast to the total health expenditure in other countries of the CIS, but most notably Georgia’s neighbour. The percentage of total expenditure on health in 2007 according to the MoLHSA is presented in Figure 4[2].

According to the national health report in 2008, the total expenditure on health as a share of GDP increased and was 8.7%. In addition, in 2008, gross domestic product increased by 1.8% of state expenditure on health [2,18-20,22]. In 2009, primary health care costs were funded by public sources for most of the rural population, and the urban population and funds or health care in 2009 was 6.9% of the whole budget.

According to the MoLHSA annual report in 2009, from the total health expenditures on health care, 39.1% have been spent on inpatient services and 21.9% have been spent on outpatient services; the total health expenditure of whole inpatient spending the 22.8% have been financed from the state sector and 69.5% from the private sector. From the point of view of outpatient services, 20.8% of expenditure was funded from the state sector and 49.5% from the private sector.

**Figure 5:**
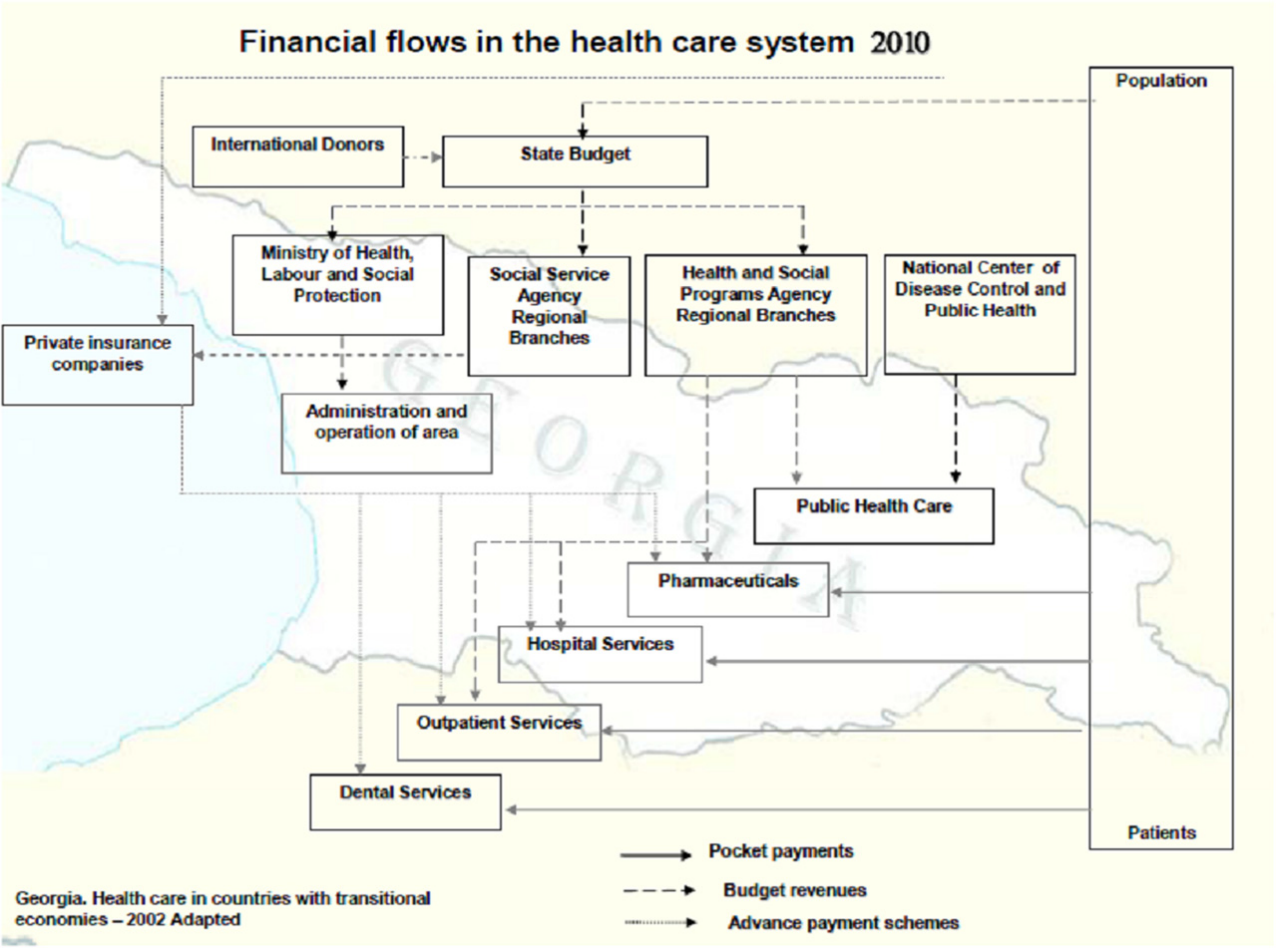
Financial flow in health care system in Georgia.

The survey of nationwide public study on medical expenses of health service delivery in 2007 showed that the country is strongly behind the international indicator of GDP. The number of requirement per person for the CIS countries is more than 8 (2007), in the European Union — 7.8 (2006), in Azerbaijan — 4.6 (2007), in Armenia — 3 (2007) and in Georgia — 2.1 (2008) [2,10,12]. Based on the literature and practice, for the first contact with the health care system, only 53% of the population elected the primary health care institutions; others were admitted in outpatient departments of the hospitals or other medical institutions. The financial flow in health care system in 2010 is presented in Figure 5.

**Figure 6:**
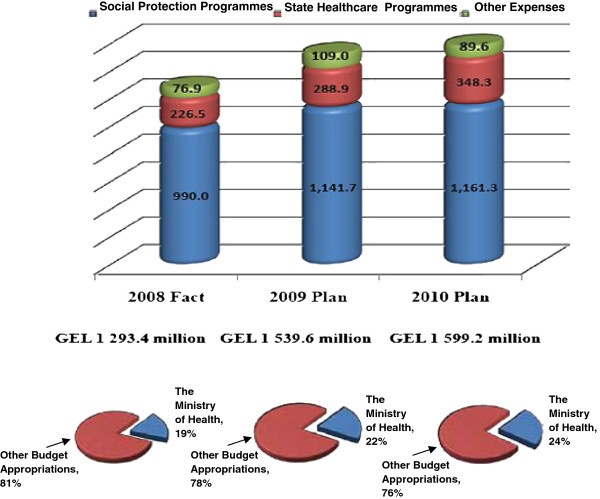
Breakdown of State Budget for Health and Social Care in 2010 (Source - A Citizen’s Guide to the 2010 State Budget of Georgia. Report of Ministry of Economics).

The breakdown of state budget for health and social care programs funded by the state programs in 2010 is presented in the Citizen’s Guide to the 2010 State Budget of Georgia—report of the Ministry of Economics and shown in Figure 6[2,13,22].

**Figure 7:**
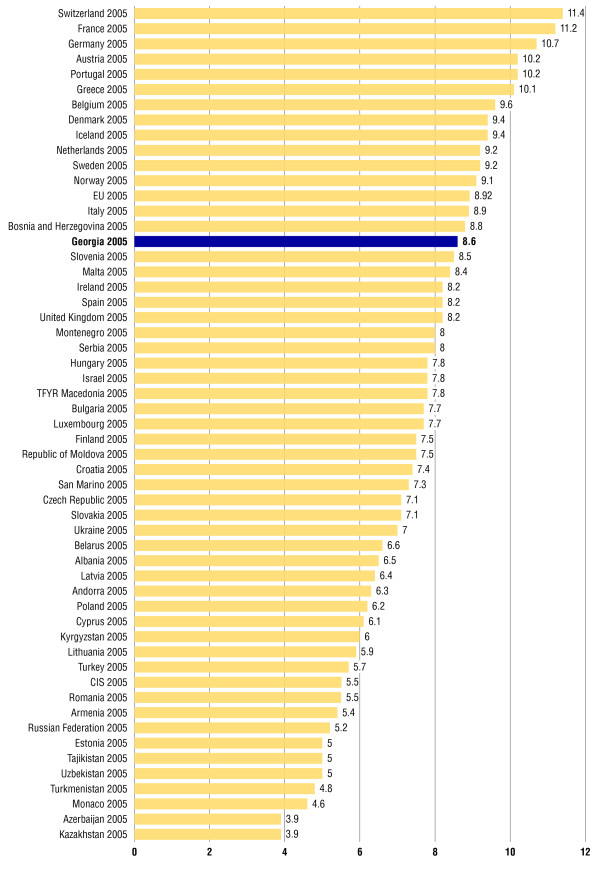
**Health expenditure as a share (%) of GDP in the WHO European Region.** Presented by WHO in 2009 taken from [2].

In 2001–2008, total expenditure on health per capita from 115 GEL has been increased to 379 GEL. Public expenditure on health care per capita has been increased from 17 to 78 GEL from the state site and from 88 to 261 GEL from the private site. The total health care expenditures have significantly increased in the past several years and reached 10.1% of the GDP in 2009, which is almost twice as high as that of the countries with a comparable economic development to Georgia’s. Health expenditure as a share (%) of GDP in the WHO European Region presented by WHO in 2009 is shown in Figure 7[20,22].

#### Human resources in health

**Figure 8:**
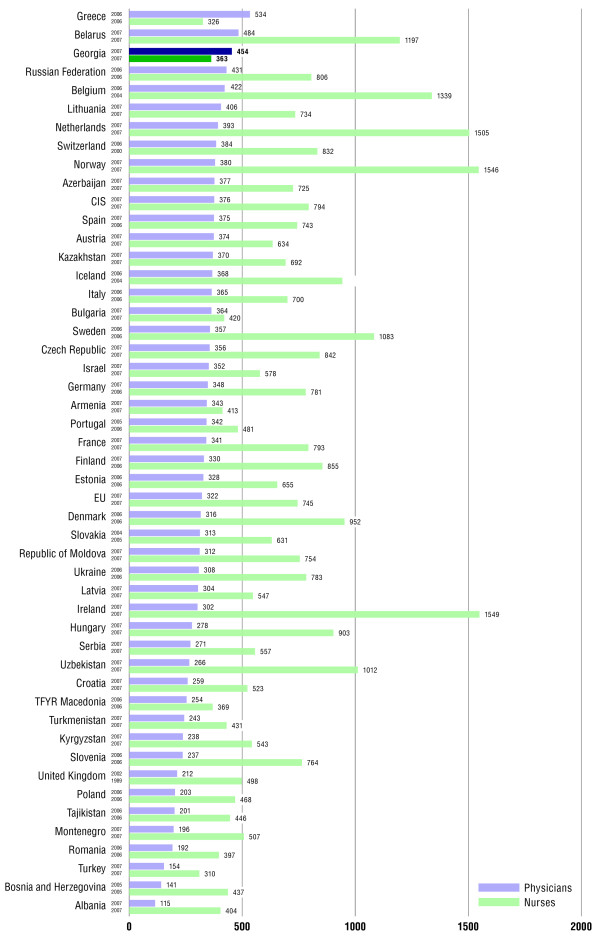
Number of physicians and nurses per 100,000 population in Georgia and selected other countries in WHO European Region. Presented by WHO in 2009 (taken from [2]).

According to all official documents represented by educational institution, national and international organisation reports, there is an extremely high number of doctors per capita in Georgia compared with other European countries [2,4,10,13,16,20]. In Georgia, the number of physicians is higher than the European average, where there are 462 doctors per 100,000 people, compared to 327 in European countries. At the same time, the country faces an acute shortage of nurses, both in urban and rural areas. The country’s educational institutions produce far more doctors than needed. Approximately 1,200 doctors (excluding dentists) enter the labour market every year, whereas only about 100 nurses graduate every year from nursing schools. Therefore, Georgia’s annual physician production count per 1,000 inhabitants is three times higher than the European average, while the number of nurses produced by the educational system is more than ten times less than observed in Europe (Figure 8).

The current system for undergraduate and postgraduate medical education does not prepare students with adequate theoretical knowledge and clinical skills, which further aggravates the human resource problems. Consequently, the quality of human resources poses significant challenges to the sector. According to the experts’ need assessment, WHO and MoLHSA reports, Georgia needs approximately 350 doctors and 1,400 nurse graduates annually to be on par with an average European country. It is imperative that medical schools plan their production volumes according to the country’s needs [2,4,10,13,20].

In 2009, the index of medical personnel providing the care was 424.9 (for medical doctors) and 134.1 (nurses). According the data of MoLHSA in recent years, the ratio of doctors to the other medical staff is not changed and is approximately 1:1, which is much less to compare than the same indicator in European countries and the WHO recommended ratio (1:4). There were 320 hospital beds per 100,000 people in 2008 and three times as many physicians in the capital city, Tbilisi, than in rural areas [2,4,6-9,18].

#### Health care delivery and recording

MoLHSA in 2009 reported the 2,216,203 recorded disease case by the outpatient services and rural health care professionals (prevalence rate 50,243.8), including the first time diagnosis, 1,169,546 (incidence rate 26,514.9). In recent years, the prevalence and incident rates have been characterised by the tendency of growth. From the registered 2,216,203 cases in 2009, 608,034 cases were recorded in rural population. This number includes the cases where the diseases have been diagnosed first time (378,563 cases). Compared to 2008, in 2009 in Georgia, the number of registered cases of diseases increased and reached 18.4%, and the first recorded incidence of diseases increased and reached 31%.

In 2009, 318,872 patients were admitted for treatment to hospitals. During the last years, the increasing rates of the hospitalisation were noted. By the end of 2009, the hospitalisation rate decreased and was 7,229.2 per 100,000 capita [4,10,12-16]. In recent years, the general trend of increasing prevalence and incidence, which is especially apparent in 2009, was noted in Georgia.

To count in real numbers, compared to 2008, general prevalence was increased from 41,270.3 to 50,243.8 and incidence from 18,420.0 up to 26,514.9. There is increased prevalence and incidence rates in children under 15 years (prevalence in 2008 was 48,946.4, in 2009 — 63,198.8, incidence in 2008 — 36,808.3, in 2009 — 52,339.1) [4,10,12-15].

#### The reform framework

##### Major health care reforms in Georgia

The development of the Primary Health Care Master Plan began in 2003 with support from the international aid sources. According to the official records and literature review, the plan was outlined to consolidate the 750 existing primary health care facilities outside of Tbilisi into 549 facilities that would serve approximately 30,000 people each. In 2006, the State Agency for Social Assistance was created, along with the Governmental Commission for Health and Social Reforms, which became the decision-making body for health care reforms. As a fact, the first policy created, entitled Main Directions in Health 2007–2009, and outlined four main health objectives for the government to address: affordability of basic health services and protection of the public from serious financial health risks, quality of services, accessibility of services by continued development of infrastructure, and efficiency of the health system.

In 2008, the Ministry of Labor, Health, and Social Affairs distributed primary health care ‘toolkits’, which included renovation plans and funds, to rural providers in about 900 rural villages. The first reform to be implemented as part of the Main Directions in Health was the Hospital Development Master Plan [2,20].

Beginning in January 2007, the reform resulted in the replacement of the existing hospital infrastructure by transferring ownership rights from the state to the private sector. Hospital locations were chosen based on the principle of 45-min geographic accessibility, with the number of beds based on population size and health needs. Newly reformed hospitals integrated psychology, necrology, oncology, obstetrics, gynaecology, paediatrics and infectious diseases meant to provide comprehensive quality health care [2,12,13,16,20].

In 2011, MoLHSA developed and represented the ‘Georgia: National Health Care Strategy 2011–2015,’ which was reviewed and evaluated during this period and clearly demonstrated the mission, vision and strategic planning of future activities. According to this document, health care system should be focused on the patients’ needs rather than their purchasing capacity. The individualisation of medicine and health care appears to be following a general societal trend. The terms ‘personalised medicine’ and ‘personal health’ are used to describe this process [11]. State subsidies for individual health care services should be focused on the patients and should ensure the freedom of choice. The document underlines that patient-focused health care system is a system that is focused on the following key values: patient’s awareness, freedom of choice, patient’s safety and protection from inefficient use of medical services[12,13,20].

The most important directions in the way of health care improvement were represented:

• *Access to quality medical care* is one of the main prerequisites for improving the health of the population and for addressing the health care challenges the nation currently faces.

• *Informing the Georgian population and medical society about the reforms* is the health care strategy planned to help ensure their active involvement during the implementation.

• Equal access to health care should serve as a safety net for all Georgian citizens, especially those living below the poverty line, residents of occupied territories, and people with disabilities, the rural population and prison inmates.

In the Georgia: National Health Care Strategy 2011–2015, the following government responsibilities were mentioned:

• Make public health care services available for all citizens of Georgia.

• Assure a competitive environment in order to secure better quality and affordable medical insurance and health care services for the population.

• Enact policies that will guarantee the protection of each citizen’s legitimate rights in the health sector.

• Underline that both the state and the private sectors are accountable to the public in the health sector. Health care resources should be adequate and sufficient to perform the tasks faced by the health care sector. The number and qualification of medical and managerial staff, financial resources and infrastructure should be requisite to meet the health needs of the nation.

Many challenges and problems currently faced by the health sector require productive cooperation between different agencies as well as the coordination of activities of the state, private and non-governmental sectors [12,13,20]. The multidisciplinary and multisectoral involvement is essential to developing PPPM interventions; multinational cooperation would, additionally, provide maximum benefit [11]. Among the different challenges which have been identified by MoLHSA, the problematic topics, which might be underlined, are as follows:

1. *Demographic challenges*. This positive trend was accompanied by an increased fertility rate, which has risen significantly over the past several years and reached 1.86 per reproductive aged women in 2010; nevertheless, it is still less than the population replacement rate of 2.15.

2. *Population health challenges* vary by different age groups. The difference in health problems of various age groups calls for differentiated preventive and curative interventions. Moreover, risks posed by existing and emerging infectious diseases call for strengthening the infectious disease surveillance system and initiating a timely and coordinated response to emerging biological risks.

3. *Health care system challenges* might be faced in health care financing, medical incurrence, health human resources, patient right protection and information technologies.

It should be noted that towards the end of 2011, fifty hospitals had been completely remodelled or constructed. Therefore, renovating and constructing new health care facilities and equipping them with modern medical equipment, as well as spatial and functional planning of a provider network must be adequately addressed in the new strategy.

The strategic objectives have been identified by the government of Georgia and MoLHSA, and the strategic initiatives to meet the main goal—reduce inequalities in access to medical care and to raise the number of insured population to at least 2.5 million by 2015—were elaborated.

In order to achieve these goals, the government of Georgia has drawn up five strategic objectives and defined 26 strategic initiatives, and all of them are described below [12,13,16,20].

According to the MoLHSA strategic document, the government will meet this objective through the implementation of the following strategic initiatives:

1. *Reduce inequalities in access to medical care.* Equal financial and geographic access to health care services is one of the primary objectives of the government.

(a) *Implementation of hospital sector development plan for 2011–2015*. Within the frames of the health infrastructure development programme, 150 medical facilities are targeted to be renovated and/or reconstructed by 2013.

(b) *Increase the number of insured citizens*. For the past 5 years, the number of insured people has increased from 40,000 to 1.5 million. As of 2011, more than one million people are covered by state-funded medical insurance, including more than 900,000 citizens living below the poverty line.

(c) *Develop primary health care.* All citizens, especially in rural areas, should have access to primary health care (PHC) services.

(d) *Improve access to medicines.* In 2009, through amendments in the ‘Law on Drugs and Pharmaceutical Activities’, the government introduced mutual recognition and parallel import regimes to reduce market entry barriers for new pharmaceuticals.

(e) *Increase access to medical care for people with special needs.* The government plans to reduce physical access barriers to medical service for people with disabilities by developing infrastructural requirements and incorporating them in permit regulations for health care establishments.

(f) *Health care in the penitentiary system.* Assuring and improving the quality of medical services is a priority objective for the government.

2. *Improve quality of medical services.* Improving the population’s health and increasing financial protection from ill health will not be possible without improved access to outpatient drugs, which will help to promote PPPM implementation in Georgia.

(a) *New permission requirements for medical institutions.* To improve the system of permits, which should define the list of minimum mandatory requirements to be met by an inpatient facility. These requirements will be geared towards assuring patients’ safety in a health care setting.

(b) *Motivated and qualified medical personnel*. Reducing the number of physicians and increasing the number of nurses are essential pre-requisites to meet the country’s needs for health care personnel.

(c) *Accreditation of medical programmes and clinical placements.* Based on an updated list of specialties and competencies for physicians, in 2013, new accreditation requirements for postgraduate programmes will be introduced.

(d) *Quality improvements in health care*. A voluntary accreditation system of medical facilities will be gradually established.

(e) *Promoting evidence-based clinical practice.* The MoLHSA, in collaboration with professional associations (which will play a leading role in the process) and health care investors, will support the development, implementation and regular update of evidence-based guidelines.

(f) *Create and develop pathology services.* It is an important prerequisite for the improvement of the quality of diagnostics and curative services, medical education and health care in general.

3. *Protect patient’s rights.* Protecting patient rights in dealing with insurance companies and medical establishments is one of the key objectives for the government. Therefore, the government plans to improve insurance regulations in order to (a) protect the interests of insured people and (b) to facilitate the expansion of the number of self-insured in the country.

(a) *Develop mediation service and electronic portal for citizens.* To consider that the citizen, the government and the private sector can obtain required information about a specific facility or personnel and make an informed decision.

4. *Prevent diseases, assure preparedness and response to health threats.* Encompass health risk assessment, bioterrorism and pandemic preparedness and response in addition to monitoring water, environment and food safety, etc. (managed by NCDCPH), having huge importance to improve, implement and promote PPPM in Georgia.

(a) *Enhance public health system*. To develop an integrated disease surveillance system, which will be equipped in accordance with modern standards, including the necessary information infrastructure, upgraded laboratory network and highly qualified personnel.

(b) *Enhance maternal and child health services.* To reduce child morbidity and mortality, the increase of effectiveness of the immunisation program by reaching and maintaining high immunisation coverage rates.

(c) *Prevention and control of tuberculosis and HIV/AIDS.* To reduce the morbidity and improve treatment outcomes.

(d) *Prevention and screening of non-communicable disease*. In order to reduce the burden caused by non-communicable diseases and improve their surveillance, chronic disease registers (NCDC) will be developed; based on which, the country will plan and implement specific preventive measures.

(e) *Support the mental health.* Increasing physical and geographical access to psychiatric services for the population of Georgia.

(f) *Health promotion and healthy lifestyle.* Health promotion combines education, prevention and measures that create a healthy environment. To promote a healthy lifestyle is to develop and implement information, education and communication initiatives aimed at increasing the population’s awareness about health and healthy lifestyle issues.

(g) *Assure emergency and disaster preparedness.* The emergence of biological and other man-made or natural hazards poses high economic and social risks in the country.

5. *Improve management of the health sector, increase efficiency.* Increasing role of the private sector in health care financing, service provision, medical education, and supply of medical equipment and medicines. In the same way, multidisciplinary and multisectoral involvement is essential to developing PPPM interventions; multinational cooperation would, additionally, provide maximum benefit [11].

(a) *Increase effectiveness of the health care system.* Evaluate the effectiveness of the health care system on an annual basis.

(b) *Implement electronic health care.* The integrated information system will acquire a new function—it will be a national registry of individual service providers that combines data about the qualifications and certifications of health care specialists.

(c) *Enhance inter-sectoral coordination mechanisms for specific objectives and support health science.* Success of the strategy largely depends on access to evidence, which can be obtained through research using routine/administrative information and data derived from special studies.

All the above-mentioned strategies and activities will be supported by MoLHSA.

## Conclusions

Georgia has made a significant effort to adapt health policy and health system to the new environment. According to the MoLHSA strategic plan, the government intends to improve population health through a reduction of disease burden and mortality by 2015 [12,13,16,20]. The way of implementing quality and patient-focused health care system is essential:

• Improve the quality of health infrastructure, where the 8,000 hospital beds will be created in scale of the country. As a result, all Georgian citizens will be able to receive high quality medical services in close proximity to their residences.

• Improve access to PHC services, and for this, the government should contribute to the functional integration of rural and district PHC facilities with other levels of medical care. Finally, PHCs should become fully integrated in the unified health information system.

• Improve the quality of monitoring systems, create and develop the pathology services and support the implementation of evidence-based clinical guidelines, which will be successful steps in the way of improving health care system in Georgia.

• Develop the quality assurance systems for outpatient and laboratory service providers.

• To achieve the proper balance between the health care professionals, which will help to avoid unjustified costs spent on human resource development, undesirable clinical outcomes and the challenges of employing an excessive number of physicians in the health sector.

• Accreditation of health care facilities and institutions and development of external quality control systems.

• Implementation of the electronic health care, which will enhance population health monitoring and health risk assessment functions. As a result, population health risks will be appropriately identified and will be possible to evaluate the quality of provided medical service.

• To improve the quality of medical services through the prevention, promotion of healthy lifestyle, protection of the patient rights, advocacy, information and involvement in self care, it is essential that each citizen be fully aware of the adverse effects of unhealthy behaviour.

• All the above mentioned will be important predictors of successful promotion of PPPM in the country. PPPM has the potential to help us deal with many of the concerns facing health care systems in relation to disease and economic burden, whether in high-, middle- or low-income countries [11].

Accordingly, all the above-mentioned activities will result to the following:

• Population’s life expectancy will rise because of reduced mortality resulting from averted premature death, primarily among children as well as other age groups.

• The quality of life will improve because of reductions in morbidity and associated disability rates.

## Competing interests

The author has no competing interests.
